# Impacts of Colored Light-Emitting Diode Illumination on the Reproductive Performance and Bioactive Constituents and the Molecular Mechanism of Hypothalamus Gland in Zi-geese

**DOI:** 10.3389/fvets.2022.874679

**Published:** 2022-04-11

**Authors:** Li Manyu, Zhao Xiuhua, Liu Guojun, Zhang Guixue

**Affiliations:** ^1^College of Animal Science and Technology, Northeast Agricultural University, Harbin, China; ^2^Institute of Animal Husbandry, Heilongjiang Academy of Agricultural Sciences, Harbin, China

**Keywords:** Zi-goose, lighting, reproduction performance, metabolome, pathogenesis

## Abstract

Goose is a seasonal breeding animal. Its reproduction is regulated by hypothalamus-pituitary-gonad axis and also affected by environmental factors such as light and location. Zi-goose is characterized with long egg-laying period and high egg-laying potential and belongs to the long-day type of seasonal breeding. In this study, the regulation mechanism of different lighting on reproductive performance of Zi-goose by using metabonomics analysis technology. In addition, 1,481 differential metabolites were screened out totally. 583 differential metabolites were identification in hypothalamus of Zi-goose. 196 differential metabolites were identification in pituitary of Zi-goose. 692 differential metabolites were identification in ovary of Zi-goose. Under red light condition for 12 h, expression of 433 differential metabolites were down-regulated and expression of 150 differential metabolites were up regulated in hypothalamus of Zi-goose, expression of 125 differential metabolites were down-regulated and expression of 71 differential metabolites were up-regulated in pituitary of Zi-goose, expression of 355 differential metabolites were down-regulated and expression of 337 differential metabolites were up-regulated in ovary of Zi-goose. 33 differential metabolites were closely associated with 1,264 transcripts and 400 homologous genes of related enzymes in hypothalamus of Zi-goose. 15 differential metabolites were closely associated with 163 transcripts and 47 homologous genes of related enzymes in pituitary of Zi-goose. 55 differential metabolites were closely associated with 1,255 transcripts and 360 homologous genes of related enzymes in ovary of Zi-goose. It was confirmed that four metabolic pathways were closely related to light regulation of reproductive performance of Zi-goose, namely GnRH signaling pathway, prolactin signaling pathway, thyroid hormone synthesis and ovarian steroidogenesis. Typical differential metabolites of arachidonic acid, glucose-6-phosphate, progesterone, glutathione, oxidized glutathione, testosterone, deoxyepiandrosterone and their related protein genes would play an important role in light regulation of reproductive performance of Zi-goose.

## Introduction

Metabonomics is an essential component of systems biology (1). Its research idea is to follow the example of genomics and proteomics, using high-throughput and high-sensitivity modern analysis technology to quantitatively analyze all metabolites in the organism as well as to find the corresponding relationship between metabolites and physiological and pathological changes (2, 3). Metabolomics is the study of all metabolites in a sample at a certain time by LC-MS/GC-MS. Metabonomics magnifies the slight variations of gene and protein expression reflecting the physiological and pathological state of the body system (4). Through metabonomics analysis, the different metabolites between the experimental group and the control group could be further clarified, which provides a linkage for revealing the material and energy metabolism activities between the experimental and the control groups (5, 6).

In the previous studies, it has been indicated that 12 h red light was beneficial to improve the reproductive performance of Zi-geese, and the differentially expressed candidate genes and possible regulatory pathways of Zi-geese under different light conditions were analyzed by transcriptome sequencing technology (7, 8). To further reveal the mechanism of the 12 h red light improving the reproductive performance of geese, the samples of the hypothalamus, ovaries, and pituitaries from the Zi-geese during the peak laying season were studied through this research and those samples processed through the metabolomics, transcriptomics, and metabolomics combined analysis. This study was aimed to find out the differential metabolites in response to light regulation, and to clarify the regulatory genes of the differential metabolites and their differences in the regulation mode of reproductive performance for the geese, as well as to further analyze the regulation mechanism of different light conditions on reproductive performance of the Zi-geese.

## Methods and Material

### The Experimental Materials

Fifty layings Zi-geese were purchased from the Yuanfang geese industry in Harbin.

### Animals Experiment

The fifty geese were randomly separated into two groups with 25 geese in each group. Those geese were respectively treated with the white incandescent lamp and red LED lamp set 12 h long. The experimental groups were shown in Table 1. Conventional feeding management, mixed group feeding of male and female geese, as well as the experimental diet, were shown in Table 2. At the end of the experiment, 3 geese in each treatment were randomly slaughtered. Thalamus, pituitary, and ovary tissues were sampled and put into an enzyme-free aseptic cryopreservation tube and quickly frozen in liquid nitrogen. After that, they were taken back to the laboratory and stored at −80°C for transcriptome and metabonomic analysis.

**Table 1:** Experimental grouping and sampling number.

**Groups**	**Treatment**	**Sampling organ**	**Serial number**
Experimental group	Red LED 12 h	Hypothalamus, hypophysis, oophoron	RH RP RO
Control group	White light 12 h	Hypothalamus, hypophysis, oophoron	WH WP WO

**Table 2:** Joint analysis results of hypothalamic metabolites and transcripts in Zi-geese.

**Metabolite ID**	**Metabolite name**	**Transcript no**.	**Homologous genes no**.
C00003	Nicotinamide adenine dinucleotide	359	116
C00019	S-adenosine methionine	218	83
C00022	Pyruvic acid	55	18
C00025	L-glutamic acid	103	30
C00072	Ascorbic acid	71	5
C00092	6-phosphoglucose	27	8
C00188	L-threonine	25	11
C00219	Arachidonic acid	66	20
C00410	Progesterone	19	5
C00491	l-cystine	2	1
C00504	Folic acid	1	1
C00523	Androsterone	6	1
C00526	Deoxyuridine	31	6
C00530	Hydroquinone	47	13
C00576	Betaine aldehyde	9	2
C00584	Prostaglandin E2	8	4
C00669	γ-glutamate kinase	47	3
C00777	Retinoic acid	38	12
C01561	Ossification diol	8	3
C02470	Yellow urea acid	24	13
C03167	Phosphonyl acetaldehyde	24	13
C03205	Deoxycorticosterone	7	4
C04742	15-hydroxyeicosaptaenoic acid	7	1
C05290	19-hydroxyandrosteno-4-ene-3, 17-dione	1	1
C05490	11-dehydrocorticosterone	14	8
C05503	17β-estradiol 3-(β-D-glucuronide)	6	1
C06104	Adipic acid	20	6
C06426	Gamma linolenic acid	9	4
C06428	Eicosapentaenoic acid	6	3
C08362	Palmitic acid	2	1
C15572	Guaiacol	1	1
C16285	Thiobenzamide	1	1
C18060	N-acetyl-α-d-galactosamine 1-phosphate	2	1

### Preparation of the Detection Samples

In total, 60 mg of tissue samples were added to 600 uL of methanol, shaken for 30 s, and centrifuged at 4°C and 12,000 r/min for 10 min to obtain the sample, which was used for the UPLC-Q/TOF-MS detection.

### Analyse of the Components by UPLC-Q/TOF-MS

The analysis of the drug-containing plasma samples was performed in an LC-30A UPLC (Shimadzu, Kyoto, Japan) coupled hybrid quadrupole time-of-flight tandem mass spectrometer (LC/MS-Triple TOFTM 5600+, AB Sciex, Concord, ON, Canada) equipped with an electrospray ionization (ESI) interface (9, 10). The chromatographic conditions were were consistent with those described in our previously published article.

### Construction of the Component-Target-Disease Network

Cytoscape V3.7.2 is an open-source software used to visualize complicated networks and integrate different types of attribute data (11). The aforementioned data were imported into Cytoscape V3.7.2, which was used to construct an active component-target-disease interaction network. The nodes indicate components or targets, and the edges indicate their relationships. The topological parameters, including “Degree”e “Closeness Centrality” and “Betweenness Centrality”e which can be used to evaluate the importance of components and targets, were calculated by Network Analyzer. The core components were screened as ligands for molecular docking based on the degree values. The degree value of a molecule represents the number of connections between the molecule and targets in the core architecture of the network.

### Construction of the Protein–Protein Interaction (PPI) Network

To analyze the interactions between the CR component targets and primary dysmenorrhea-related targets, the overlapping targets were imported to the STRING database (https://string-db.org/, ver. 11.0). The organism was programmed to be “*Anas*”n and the confidence level was set as “highest confidence (0.900)”0 The PPI network was obtained from the targets most strongly associated with the overlapping targets. Cytoscape V3.7.2 was used to construct and visualize the PPI network. The top 10 correlated targets were identified by calculating the correlation degree of the target proteins in the PPI network.

### GO and KEGG Enrichment Analyses

OmicShare (http://www.omicshare.com/tools) was used to visualize the results of Gene Ontology (GO) functional enrichment analysis and Kyoto Encyclopedia of Genes and Genomes (KEGG) pathway enrichment analysis (12). GO analysis included biological process (BP), molecular function (MF), and cellular component (CC) ontologies. The statistical significance threshold was set at P<0.05.

## Results

### The LC-MS Analysis of the Organ Metabolites

**Figure 1:**
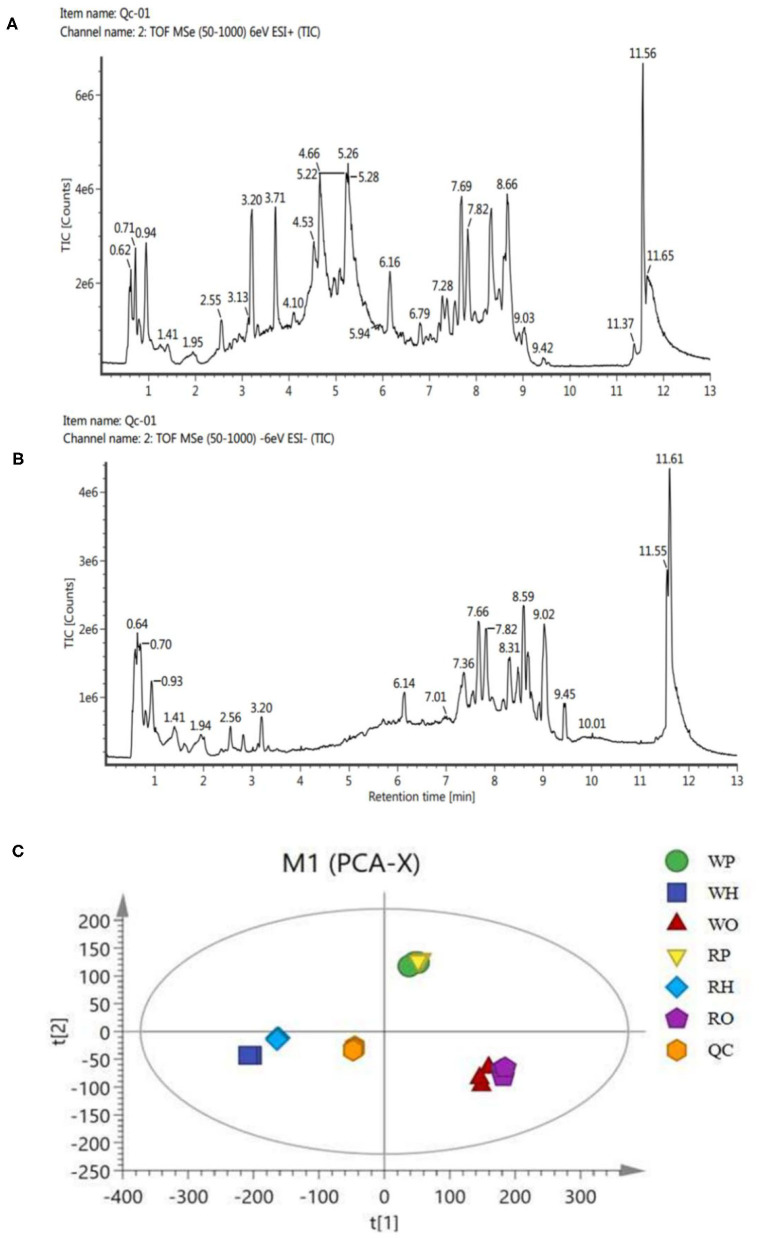
**(A)** BPI(+) ion flow chart of control samples. **(B)** BPI(-) ion flow chart of control samples. **(C)** PCA plots for all samples.

Ultra-high performance liquid chromatography-tandem time of flight mass spectrometry (UPLC-Q-TOF/MS) of Waters was used for LC-MS analysis of samples, and quality control samples were used to test the performance of the method (13). According to the ion flow diagram of the base peak of the quality control sample in the positive and negative ion mode (Figures 1A,B), it can be known that the chromatographic peak was uniform, the number was large, the peak shape was good, there was no oversaturation tailing and other adverse phenomena, and the methodology and sample quality were qualified. PCA examination of the test results (Figure 1C) showed that all the points of the quality control sample (QC) were closely clustered, indicating that the whole experimental process has been well repeated and no abnormal data.

### Identification of Metabolites and Screening of Differential Metabolites

**Figure 2:**
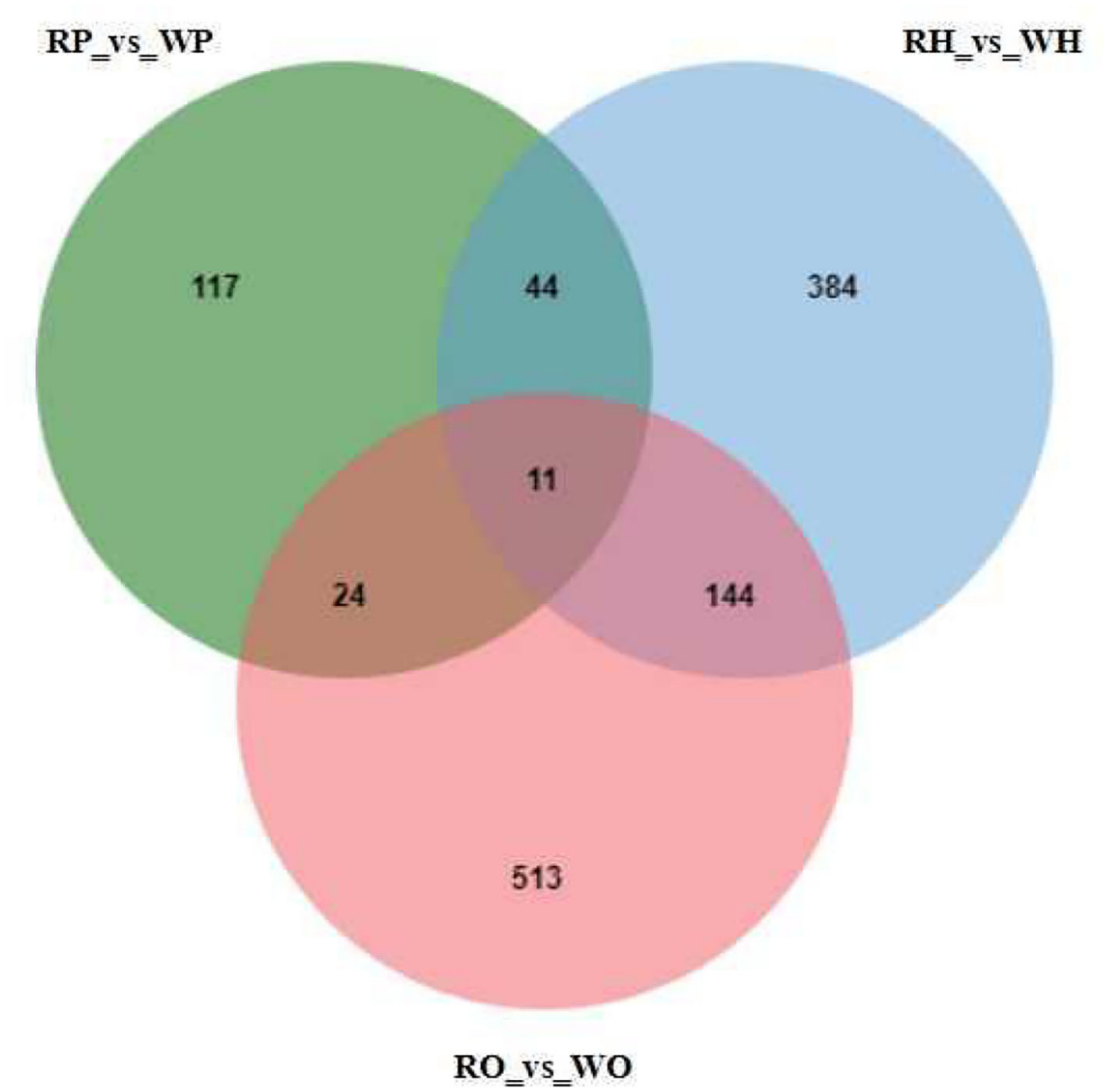
Mutual and specific differential metabolites among the difference analysis of each group.

As shown in Figure 2, 4,105 metabolites were identified based on the standard of Progenesis QI. On this basis, univariate statistical analysis was used to screen differential metabolites according to the difference multiple (FC > 2 or FC < 0.5) and *P-*value (*P-*value < 0.05, t-test). Through the experiment, 1481 differential metabolites were screened, among which 583 were screened from the hypothalamus of the candidate geese and 384 were unique; 196 differential metabolites and 117 unique differential metabolites were screened from the pituitaries of that; 692 differential metabolites and 513 unique differential metabolites were screened from the ovary of the Zi-geese. Additionally, 44 different metabolites were identification in the hypothalamus and pituitaries, 144 different metabolites were identification in the hypothalamus and ovary, 24 different metabolites were identification in pituitary and ovary, and 11 differential metabolites in the hypothalamus, pituitaries, and ovary.

### Cluster Analysis of Differential Metabolites

**Figure 3:**
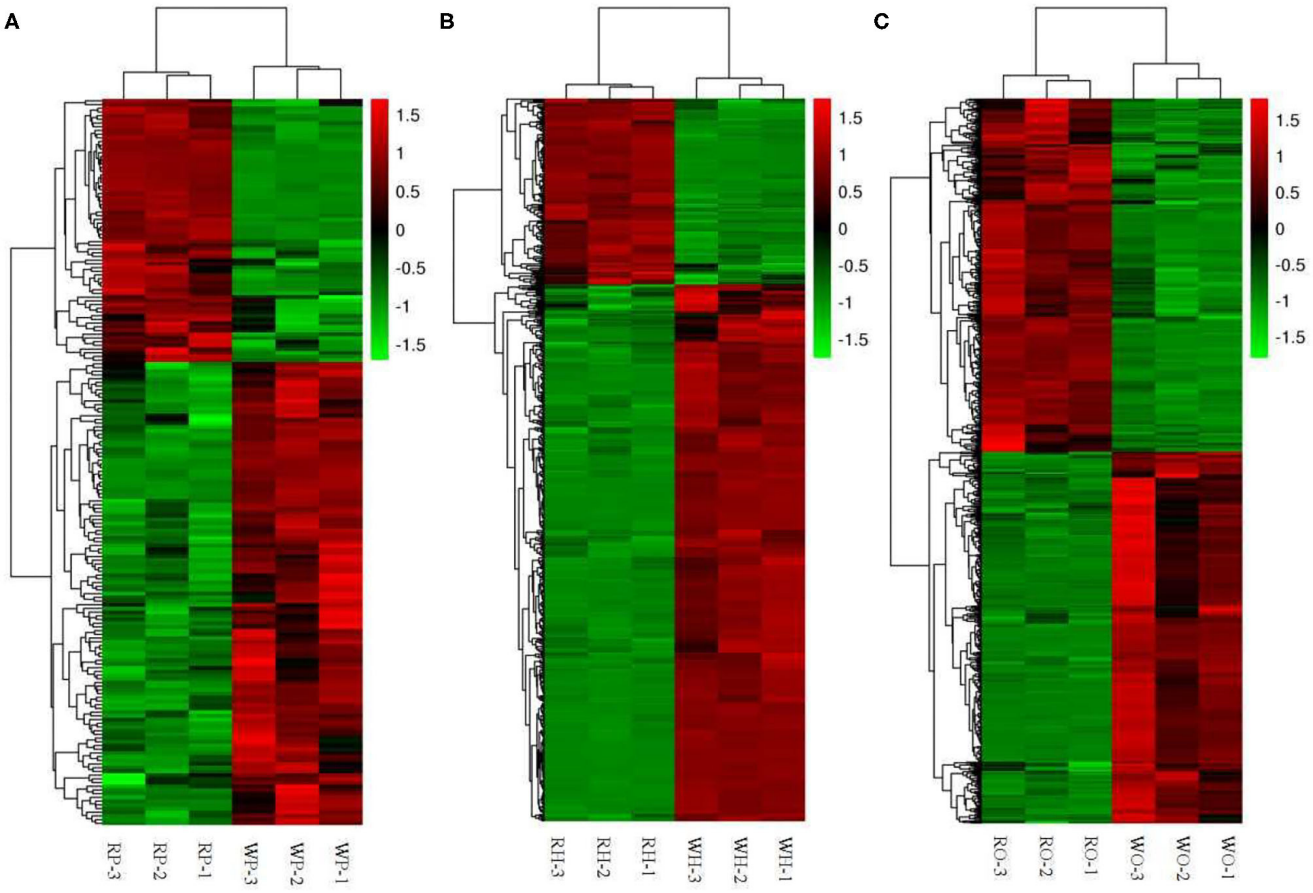
Cluster thermogram of differential metabolites. **(A)** pituitaries, **(B)** hypothalamus, **(C)** ovary.

According to the Cluster thermogram of differential metabolites, the accumulation of 125 differential metabolites in the pituitaries of the Zi-geese decreased under the 12 h red light, while the accumulation of 71 differential metabolites increased under the red light within 12 h (Figure 3A).The accumulation of 433 differential metabolites in the hypothalamus of the geese decreased under the light, while 150 differential metabolites increased under the same condition(Figure 3B). The accumulation of 355 differential metabolites in the ovary of the geese decreased under the 12 h red light, and 337 differential metabolites increased under the red light (Figure 3C). The accumulation of these differential metabolites in different organs will be the main basis for the improvement of reproductive performance of the Zi-geese under red light for 12 h.

### Combined Analysis of Metabolites and Transcripts

The results of the combined analysis showed that 33 differential metabolites were closely related to 1,264 transcripts and 400 homologous genes of related enzymes in the hypothalamus of the geese (Table 2); In the Zi-geese' pituitaries, 15 differential metabolites were closely related to 163 transcripts and 47 homologous genes of related enzymes (Table 3); There were 55 differential metabolites in Zi-geese' ovary, which were closely related to 1,255 transcripts and 360 homologous genes of related enzymes (Table 4).

**Table 3:** Joint analysis results of pituitary metabolites and transcripts in Zi-geese.

**Metabolite ID**	**Metabolite name**	**Transcript no**.	**Homologous genes no**.
C00036	Butanone diate	44	12
C00158	Citric acid	15	3
C00490	Itaconic acid	2	1
C00534	Pyridoxamine	9	4
C01561	Ossification diol	8	3
C02166	Leukotriene C4	2	1
C05422	Dehydroascorbic acid	8	3
C05584	Vanilla aldehyde acid	10	2
C05587	3-methoxytyramine	4	2
C06104	Adipic acid	20	6
C06427	Alpha linolenic acid	34	7
C15658	6-(α-D-glucose−1-D-inositol)	4	1
C16702	4 '-o-methyl norbornene	1	1
C16836	1, 4 '-piperidin-1' -carboxylic acid	2	1

**Table 4:** Joint analysis results of ovarian metabolites and transcripts in Zi-geese.

**Metabolite ID**	**Metabolite name**	**Transcript no**.	**Homologous genes no**.
C00037	Glycine	56	21
C00049	L-aspartic acid	79	11
C00051	Glutathione	78	21
C00065	L-serine	20	7
C00072	Ascorbic acid	71	5
C00073	L-methionine	54	24
C00074	Phosphoenolpyruvate	16	4
C00097	L-cysteine	93	14
C00120	Biotin	15	5
C00127	Oxidized glutathione	15	4
C00143	5, 10-methylene–tetrahydrofolate	24	7
C00153	Nicotinamide	64	20
C00160	Glycolic acid	5	1
C00198	Gluconolactone	2	1
C00213	Creatine	8	3
C00248	Sulfur symplectic amide	7	1
C00253	Niacin	4	2
C00262	Hypoxanthine	7	3
C00294	Inosine	25	4
C00314	Pyridoxine	9	4
C00366	Uric acid	3	1
C00378	Thiamine	28	5
C00385	Xanthine	9	4
C00386	Carnosine	9	4
C00387	Guanosine	22	2
C00491	l-Cystine	2	1
C00530	Hydroquinone	47	13
C00535	Testosterone	43	14
C00576	Betaine aldehyde	9	2
C00581	Guanidine acetic acid	5	2
C00628	Gentisic acid	2	1
C00735	Cortisol	30	11
C00881	DNA cytidine	24	3
C00954	Indole acetic acid	24	8
C01081	Thiamine single	22	12
C01157	hydroxyproline	14	3
C01227	DHEa	23	11
C01561	Ossification diol	8	3
C01762	Xanthine nucleoside	25	3
C01879	The focal glutamate	43	3
C02029	Dihydrozealin	40	20
C02067	pseudouridine	1	1
C02137	Phenylglyoxylic acid	12	7
C05443	Vitamin D3	3	1
C05512	DNA inosine	7	4
C05582	Homovanillic acid	10	2
C05585	2, 5-dihydroxybenzaldehyde	2	1
C05903	As the alcohol	40	20
C06104	Adipic acid	20	6
C06427	Alpha linolenic acid	34	7
C06672	Vanillic acid	24	13
C15572	Guaiacol	1	1
C15658	6-(α-D-glucose−1-D-inositol)	4	1
C16285	Thiobenzamide	1	1
C17232	2-oxo-10-methyl thio-decanoic acid	12	7

According to the results of differential expression between metabolites and corresponding transcripts (log2 (FC) > 1), 15 pairs of metabolites were further screened in the hypothalamus of Zi-geese. The co-expression of transcripts significantly responded to the changes of light conditions (Table 5), and the marker metabolites were nicotinamide adenine dinucleotide, pyruvate, L-glutamic acid, ascorbic acid, 6-phosphate glucose ester, arachidonic acid, prostaglandin E2, glutamic acid γ-Glutamate kinase and hydroquinone. Three pairs of metabolites were further screened out from the pituitary gland of Zi-geese. The co-expression of transcripts significantly responded to the changes of light conditions, and the marker metabolites involved were vanillic acid and adipic acid (Table 6). 186 pairs of metabolites were further screened out in the ovary of Zi-geese. The co-expression of transcripts significantly responded to the changes of light conditions, involving marker metabolites including glycine, L-aspartic acid, glutathione, L-serine, ascorbic acid, L-methionine, phosphoenolpyruvate, L-cysteine, oxidized glutathione, 5,10-methylenetetrahydrofolate, nicotinamide, glycolic acid, gluconolactone, sarcosine, nicotinic acid, hypoxanthine Inosine, pyridoxine, thiamine, xanthine, carnosine, guanosine, L-cystine, hydroquinone, testosterone, betaine aldehyde, guanidino acetic acid, gentian acid, cortisol, deoxycytidine, thiamine monohydrate, hydroxyproline, dehydroepiandrosterone, ossifying diol, xanthine nucleoside, pyroglutamine, dihydrozeatin, phenyl glyoxylic acid, deoxyinosine, 2,5-dihydroxybenzaldehyde, canfel Adipic acid α- Linolenic acid, hydroquinone, 6-( α- D-glucose-1-d-inositol) and 2-oxo-10-methylthiodecanoic acid (Table 7).

**Table 5:** Results of differential co-expression of hypothalamic metabolites and related transcripts in Zi-geese.

**Metabolite_Transcript ID**	**Type**	**log2(FC)**
C00003_TRINITY_DN136565_c0_g1	Metabolics	1.08
C00003_TRINITY_DN136565_c0_g1	mRNA	−1.79
C00003_TRINITY_DN127160_c0_g1	Metabolics	1.08
C00003_TRINITY_DN127160_c0_g1	mRNA	1.09
C00003_TRINITY_DN119684_c1_g3	Metabolics	1.08
C00003_TRINITY_DN119684_c1_g3	mRNA	−1.42
C00003_TRINITY_DN121542_c0_g4	Metabolics	1.08
C00003_TRINITY_DN121542_c0_g4	mRNA	1.22
C00003_TRINITY_DN134625_c3_g2	Metabolics	1.08
C00003_TRINITY_DN134625_c3_g2	mRNA	−2.14
C00022_TRINITY_DN119684_c1_g3	Metabolics	−1.26
C00022_TRINITY_DN119684_c1_g3	mRNA	−1.42
C00022_TRINITY_DN136565_c0_g1	Metabolics	−1.26
C00022_TRINITY_DN136565_c0_g1	mRNA	−1.79
C00025_TRINITY_DN129572_c3_g1	Metabolics	1.34
C00025_TRINITY_DN129572_c3_g1	mRNA	−2.56
C00072_TRINITY_DN133392_c0_g5	Metabolics	6.85
C00072_TRINITY_DN133392_c0_g5	mRNA	−4.66
C00092_TRINITY_DN126050_c0_g3	Metabolics	1.36
C00092_TRINITY_DN126050_c0_g3	mRNA	−2.15
C00219_TRINITY_DN122928_c1_g1	Metabolics	−1.23
C00219_TRINITY_DN122928_c1_g1	mRNA	−1.16
C00584_TRINITY_DN134625_c3_g2	Metabolics	−1.43
C00584_TRINITY_DN134625_c3_g2	mRNA	−2.14
C00669_TRINITY_DN113290_c0_g1	Metabolics	1.74
C00669_TRINITY_DN113290_c0_g1	mRNA	1.44
C15603_TRINITY_DN121542_c0_g4	Metabolics	−1.00
C15603_TRINITY_DN121542_c0_g4	mRNA	1.22

**Table 6:** Results of differential co-expression of pituitary metabolites and related transcripts in Zi-geese.

**Metabolite_Transcript ID**	**Type**	**log2(FC)**
C05584_TRINITY_DN136141_c0_g1	Metabolics	−1.07
C05584_TRINITY_DN136141_c0_g1	mRNA	−4.10
C05584_TRINITY_DN130821_c0_g3	Metabolics	−1.07
C05584_TRINITY_DN130821_c0_g3	mRNA	−4.99
C06104_TRINITY_DN139541_c0_g1	Metabolics	−2.85
C06104_TRINITY_DN139541_c0_g1	mRNA	−1.40

**Table 7:** Results of differential co-expression of ovarian metabolites and related transcripts in Zi-geese.

**Metabolite_Transcript ID**	**Type**	**log2(FC)**
C00037_TRINITY_DN129582_c7_g1	Metabolics	−1.85
C00037_TRINITY_DN129582_c7_g1	mRNA	1.13
C00037_TRINITY_DN137486_c6_g2	Metabolics	−1.85
C00037_TRINITY_DN137486_c6_g2	mRNA	1.08
C00037_TRINITY_DN111806_c0_g1	Metabolics	−1.85
C00037_TRINITY_DN111806_c0_g1	mRNA	−8.97
C00037_TRINITY_DN118954_c0_g3	Metabolics	−1.85
C00037_TRINITY_DN118954_c0_g3	mRNA	4.58
C00037_TRINITY_DN125224_c0_g1	Metabolics	−1.85
C00037_TRINITY_DN125224_c0_g1	mRNA	2.14
C00037_TRINITY_DN123719_c0_g1	Metabolics	−1.85
C00037_TRINITY_DN123719_c0_g1	mRNA	6.49
C00037_TRINITY_DN124585_c0_g1	Metabolics	−1.85
C00037_TRINITY_DN124585_c0_g1	mRNA	1.44
C00037_TRINITY_DN118954_c0_g1	Metabolics	−1.85
C00037_TRINITY_DN118954_c0_g1	mRNA	3.22
C00037_TRINITY_DN68105_c0_g1	Metabolics	−1.85
C00037_TRINITY_DN68105_c0_g1	mRNA	2.85
C00037_TRINITY_DN126075_c1_g1	Metabolics	−1.85
C00037_TRINITY_DN126075_c1_g1	mRNA	1.67
C00049_TRINITY_DN122295_c3_g1	Metabolics	−1.21
C00049_TRINITY_DN122295_c3_g1	mRNA	1.86
C00049_TRINITY_DN124466_c2_g1	Metabolics	−1.21
C00049_TRINITY_DN124466_c2_g1	mRNA	3.28
C00049_TRINITY_DN133015_c5_g3	Metabolics	−1.21
C00049_TRINITY_DN133015_c5_g3	mRNA	−1.68
C00049_TRINITY_DN136189_c6_g8	Metabolics	−1.21
C00049_TRINITY_DN136189_c6_g8	mRNA	8.87
C00049_TRINITY_DN126078_c0_g2	Metabolics	−1.21
C00049_TRINITY_DN126078_c0_g2	mRNA	1.81
C00049_TRINITY_DN141027_c3_g1	Metabolics	−1.21
C00049_TRINITY_DN141027_c3_g1	mRNA	1.73
C00049_TRINITY_DN120188_c0_g1	Metabolics	−1.21
C00049_TRINITY_DN120188_c0_g1	mRNA	−1.26
C00049_TRINITY_DN136146_c4_g1	Metabolics	−1.21
C00049_TRINITY_DN136146_c4_g1	mRNA	1.12
C00049_TRINITY_DN133015_c5_g1	Metabolics	−1.21
C00049_TRINITY_DN133015_c5_g1	mRNA	−1.53
C00049_TRINITY_DN128440_c1_g1	Metabolics	−1.21
C00049_TRINITY_DN128440_c1_g1	mRNA	2.73
C00049_TRINITY_DN132073_c2_g3	Metabolics	−1.21
C00049_TRINITY_DN132073_c2_g3	mRNA	1.14
C00049_TRINITY_DN126544_c1_g1	Metabolics	−1.21
C00049_TRINITY_DN126544_c1_g1	mRNA	1.74
C00051_TRINITY_DN128264_c3_g1	Metabolics	−3.08
C00051_TRINITY_DN128264_c3_g1	mRNA	1.71
C00051_TRINITY_DN126607_c2_g2	Metabolics	−3.08
C00051_TRINITY_DN126607_c2_g2	mRNA	1.35
C00051_TRINITY_DN126075_c1_g1	Metabolics	−3.08
C00051_TRINITY_DN126075_c1_g1	mRNA	1.67
C00051_TRINITY_DN137420_c3_g2	Metabolics	−3.08
C00051_TRINITY_DN137420_c3_g2	mRNA	−1.65
C00065_TRINITY_DN68105_c0_g1	Metabolics	−2.79
C00065_TRINITY_DN68105_c0_g1	mRNA	2.85
C00065_TRINITY_DN131715_c0_g1	Metabolics	−2.79
C00065_TRINITY_DN131715_c0_g1	mRNA	3.69
C00065_TRINITY_DN141180_c5_g1	Metabolics	−2.79
C00065_TRINITY_DN141180_c5_g1	mRNA	2.39
C00065_TRINITY_DN119556_c3_g3	Metabolics	−2.79
C00065_TRINITY_DN119556_c3_g3	mRNA	−1.19
C00072_TRINITY_DN130784_c0_g1	Metabolics	4.58
C00072_TRINITY_DN130784_c0_g1	mRNA	8.62
C00072_TRINITY_DN133392_c0_g5	Metabolics	4.58
C00072_TRINITY_DN133392_c0_g5	mRNA	−5.36
C00072_TRINITY_DN122008_c2_g1	Metabolics	4.58
C00072_TRINITY_DN122008_c2_g1	mRNA	−2.02
C00072_TRINITY_DN122360_c0_g1	Metabolics	4.58
C00072_TRINITY_DN122360_c0_g1	mRNA	−2.71
C00072_TRINITY_DN123314_c3_g1	Metabolics	4.58
C00072_TRINITY_DN123314_c3_g1	mRNA	1.28
C00072_TRINITY_DN133392_c0_g8	Metabolics	4.58
C00072_TRINITY_DN133392_c0_g8	mRNA	−10.88
C00072_TRINITY_DN120532_c0_g1	Metabolics	4.58
C00072_TRINITY_DN120532_c0_g1	mRNA	−4.28
C00072_TRINITY_DN112795_c0_g1	Metabolics	4.58
C00072_TRINITY_DN112795_c0_g1	mRNA	6.68
C00073_TRINITY_DN117575_c4_g8	Metabolics	−1.50
C00073_TRINITY_DN117575_c4_g8	mRNA	−4.78
C00073_TRINITY_DN133322_c1_g1	Metabolics	−1.50
C00073_TRINITY_DN133322_c1_g1	mRNA	1.90
C00073_TRINITY_DN117575_c4_g1	Metabolics	−1.50
C00073_TRINITY_DN117575_c4_g1	mRNA	−5.29
C00073_TRINITY_DN125846_c0_g1	Metabolics	−1.50
C00073_TRINITY_DN125846_c0_g1	mRNA	8.65
C00073_TRINITY_DN128420_c3_g1	Metabolics	−1.50
C00073_TRINITY_DN128420_c3_g1	mRNA	1.37
C00074_TRINITY_DN135685_c1_g2	Metabolics	−3.56
C00074_TRINITY_DN135685_c1_g2	mRNA	2.39
C00074_TRINITY_DN135685_c1_g1	Metabolics	−3.56
C00074_TRINITY_DN135685_c1_g1	mRNA	8.43
C00074_TRINITY_DN131703_c0_g1	Metabolics	−3.56
C00074_TRINITY_DN131703_c0_g1	mRNA	6.69
C00074_TRINITY_DN132519_c0_g3	Metabolics	−3.56
C00074_TRINITY_DN132519_c0_g3	mRNA	3.75
C00074_TRINITY_DN138024_c0_g1	Metabolics	−3.56
C00074_TRINITY_DN138024_c0_g1	mRNA	−1.54
C00097_TRINITY_DN139025_c5_g1	Metabolics	1.79
C00097_TRINITY_DN139025_c5_g1	mRNA	−2.28
C00097_TRINITY_DN141059_c4_g2	Metabolics	1.79
C00097_TRINITY_DN141059_c4_g2	mRNA	−2.68
C00097_TRINITY_DN125224_c0_g1	Metabolics	1.79
C00097_TRINITY_DN125224_c0_g1	mRNA	2.14
C00097_TRINITY_DN122396_c1_g2	Metabolics	1.79
C00097_TRINITY_DN122396_c1_g2	mRNA	2.75
C00097_TRINITY_DN139749_c0_g1	Metabolics	1.79
C00097_TRINITY_DN139749_c0_g1	mRNA	1.50
C00127_TRINITY_DN126607_c2_g2	Metabolics	−9.13
C00127_TRINITY_DN126607_c2_g2	mRNA	1.35
C00143_TRINITY_DN124585_c0_g1	Metabolics	−1.03
C00143_TRINITY_DN124585_c0_g1	mRNA	1.44
C00143_TRINITY_DN115258_c0_g1	Metabolics	−1.03
C00143_TRINITY_DN115258_c0_g1	mRNA	1.67
C00143_TRINITY_DN111951_c0_g2	Metabolics	−1.03
C00143_TRINITY_DN111951_c0_g2	mRNA	1.57
C00143_TRINITY_DN68105_c0_g1	Metabolics	−1.03
C0043_TRINITY_DN68105_c0_g1	mRNA	2.85
C00143_TRINITY_DN111951_c0_g1	Metabolics	−1.03
C00143_TRINITY_DN111951_c0_g1	mRNA	−1.73
C00153_TRINITY_DN139949_c1_g2	Metabolics	−3.73
C00153_TRINITY_DN139949_c1_g2	mRNA	−2.04
C00153_TRINITY_DN125347_c0_g1	Metabolics	−3.73
C00153_TRINITY_DN125347_c0_g1	mRNA	1.79
C00153_TRINITY_DN130743_c0_g1	Metabolics	−3.73
C00153_TRINITY_DN130743_c0_g1	mRNA	2.43
C00153_TRINITY_DN116220_c0_g1	Metabolics	−3.73
C00153_TRINITY_DN116220_c0_g1	mRNA	−1.26
C00153_TRINITY_DN133137_c0_g1	Metabolics	−3.73
C00153_TRINITY_DN133137_c0_g1	mRNA	3.16
C00160_TRINITY_DN136176_c0_g1	Metabolics	1.04
C00160_TRINITY_DN136176_c0_g1	mRNA	1.06
C00160_TRINITY_DN110820_c0_g2	Metabolics	1.04
C00160_TRINITY_DN110820_c0_g2	mRNA	6.89
C00198_TRINITY_DN123462_c0_g2	Metabolics	−9.71
C00198_TRINITY_DN123462_c0_g2	mRNA	5.29
C00198_TRINITY_DN127850_c1_g1	Metabolics	−9.71
C00198_TRINITY_DN127850_c1_g1	mRNA	5.47
C00213_TRINITY_DN111806_c0_g1	Metabolics	−1.40
C00213_TRINITY_DN111806_c0_g1	mRNA	−8.97
C00213_TRINITY_DN111951_c0_g1	Metabolics	−1.40
C00213_TRINITY_DN111951_c0_g1	mRNA	−1.73
C00213_TRINITY_DN111951_c0_g2	Metabolics	−1.40
C00213_TRINITY_DN111951_c0_g2	mRNA	1.57
C00253_TRINITY_DN130743_c0_g1	Metabolics	6.06
C00253_TRINITY_DN130743_c0_g1	mRNA	2.43
C00262_TRINITY_DN130743_c0_g1	Metabolics	−1.95
C00262_TRINITY_DN130743_c0_g1	mRNA	2.43
C00262_TRINITY_DN125813_c1_g5	Metabolics	−1.95
C00262_TRINITY_DN125813_c1_g5	mRNA	5.81
C00262_TRINITY_DN129874_c1_g1	Metabolics	−1.95
C00262_TRINITY_DN129874_c1_g1	mRNA	5.01
C00294_TRINITY_DN130743_c0_g1	Metabolics	−2.63
C00294_TRINITY_DN130743_c0_g1	mRNA	2.43
C00294_TRINITY_DN115131_c0_g1	Metabolics	−2.63
C00294_TRINITY_DN115131_c0_g1	mRNA	−1.78
C00294_TRINITY_DN137643_c0_g1	Metabolics	−2.63
C00294_TRINITY_DN137643_c0_g1	mRNA	1.24
C00294_TRINITY_DN135736_c0_g1	Metabolics	−2.63
C00294_TRINITY_DN135736_c0_g1	mRNA	4.02
C00314_TRINITY_DN141165_c7_g3	Metabolics	1.15
C00314_TRINITY_DN141165_c7_g3	mRNA	3.57
C00314_TRINITY_DN141165_c7_g1	Metabolics	1.15
C00314_TRINITY_DN141165_c7_g1	mRNA	1.63
C00378_TRINITY_DN117569_c9_g1	Metabolics	4.34
C00378_TRINITY_DN117569_c9_g1	mRNA	10.38
C00378_TRINITY_DN127457_c1_g1	Metabolics	4.34
C00378_TRINITY_DN127457_c1_g1	mRNA	1.39
C00378_TRINITY_DN127457_c1_g3	Metabolics	4.34
C00378_TRINITY_DN127457_c1_g3	mRNA	1.75
C00378_TRINITY_DN134236_c3_g1	Metabolics	4.34
C00378_TRINITY_DN134236_c3_g1	mRNA	1.13
C00385_TRINITY_DN130743_c0_g1	Metabolics	1.51
C00385_TRINITY_DN130743_c0_g1	mRNA	2.43
C00385_TRINITY_DN135376_c2_g1	Metabolics	1.51
C00385_TRINITY_DN135376_c2_g1	mRNA	1.52
C00386_TRINITY_DN139951_c1_g1	Metabolics	0.11
C00386_TRINITY_DN139951_c1_g1	mRNA	3.02
C00386_TRINITY_DN139951_c1_g4	Metabolics	1.11
C00386_TRINITY_DN139951_c1_g4	mRNA	3.12
C00387_TRINITY_DN135736_c0_g1	Metabolics	−1.83
C00387_TRINITY_DN135736_c0_g1	mRNA	4.02
C00387_TRINITY_DN130743_c0_g1	Metabolics	−1.83
C00387_TRINITY_DN130743_c0_g1	mRNA	2.43
C00387_TRINITY_DN137643_c0_g1	Metabolics	−1.83
C00387_TRINITY_DN137643_c0_g1	mRNA	1.24
C00387_TRINITY_DN115131_c0_g1	Metabolics	−1.83
C00387_TRINITY_DN115131_c0_g1	mRNA	−1.78
C00491_TRINITY_DN122396_c1_g2	Metabolics	2.80
C00491_TRINITY_DN122396_c1_g2	mRNA	2.75
C00530_TRINITY_DN138536_c1_g3	Metabolics	−7.99
C00530_TRINITY_DN138536_c1_g3	mRNA	−1.47
C00530_TRINITY_DN132747_c2_g3	Metabolics	−7.99
C00530_TRINITY_DN132747_c2_g3	mRNA	3.25
C00530_TRINITY_DN133363_c0_g1	Metabolics	−7.99
C00530_TRINITY_DN133363_c0_g1	mRNA	−2.00
C00530_TRINITY_DN137025_c2_g1	Metabolics	−7.99
C00530_TRINITY_DN137025_c2_g1	mRNA	−1.01
C00530_TRINITY_DN93937_c0_g2	Metabolics	−7.99
C00530_TRINITY_DN93937_c0_g2	mRNA	3.09
C00535_TRINITY_DN120919_c7_g2	Metabolics	1.04
C00535_TRINITY_DN120919_c7_g2	mRNA	7.52
C00535_TRINITY_DN129814_c0_g1	Metabolics	1.04
C00535_TRINITY_DN129814_c0_g1	mRNA	1.55
C00535_TRINITY_DN119253_c3_g1	Metabolics	1.04
C00535_TRINITY_DN119253_c3_g1	mRNA	5.13
C00535_TRINITY_DN129783_c0_g2	Metabolics	1.04
C00535_TRINITY_DN129783_c0_g2	mRNA	3.35
C00535_TRINITY_DN130231_c2_g1	Metabolics	1.04
C00535_TRINITY_DN130231_c2_g1	mRNA	6.55
C00535_TRINITY_DN134262_c1_g1	Metabolics	1.04
C00535_TRINITY_DN134262_c1_g1	mRNA	1.93
C00535_TRINITY_DN129783_c0_g3	Metabolics	1.04
C00535_TRINITY_DN129783_c0_g3	mRNA	2.51
C00535_TRINITY_DN134262_c0_g2	Metabolics	1.04
C00535_TRINITY_DN134262_c0_g2	mRNA	2.60
C00535_TRINITY_DN134262_c1_g2	Metabolics	1.04
C00535_TRINITY_DN134262_c1_g2	mRNA	2.24
C00535_TRINITY_DN129528_c2_g1	Metabolics	1.04
C00535_TRINITY_DN129528_c2_g1	mRNA	−3.03
C00535_TRINITY_DN133519_c0_g1	Metabolics	1.04
C00535_TRINITY_DN133519_c0_g1	mRNA	6.67
C00535_TRINITY_DN136732_c0_g3	Metabolics	1.04
C00535_TRINITY_DN136732_c0_g3	mRNA	2.44
C00535_TRINITY_DN120617_c0_g2	Metabolics	1.04
C00535_TRINITY_DN120617_c0_g2	mRNA	2.67
C00535_TRINITY_DN129783_c0_g1	Metabolics	1.04
C00535_TRINITY_DN129783_c0_g1	mRNA	2.23
C00576_TRINITY_DN126727_c0_g2	Metabolics	1.04
C00576_TRINITY_DN126727_c0_g2	mRNA	2.24
C00576_TRINITY_DN117587_c2_g1	Metabolics	1.04
C00576_TRINITY_DN117587_c2_g1	mRNA	1.90
C00576_TRINITY_DN126727_c0_g1	Metabolics	1.04
C00576_TRINITY_DN126727_c0_g1	mRNA	7.77
C00581_TRINITY_DN137240_c0_g1	Metabolics	−7.01
C00581_TRINITY_DN137240_c0_g1	mRNA	1.78
C00581_TRINITY_DN118954_c0_g1	Metabolics	−7.01
C00581_TRINITY_DN118954_c0_g1	mRNA	3.22
C00581_TRINITY_DN118954_c0_g3	Metabolics	−7.01
C00581_TRINITY_DN118954_c0_g3	mRNA	4.58
C00581_TRINITY_DN137240_c0_g2	Metabolics	−7.01
C00581_TRINITY_DN137240_c0_g2	mRNA	2.17
C00628_TRINITY_DN128233_c1_g1	Metabolics	−8.05
C00628_TRINITY_DN128233_c1_g1	mRNA	3.13
C00628_TRINITY_DN137617_c1_g1	Metabolics	−8.05
C00628_TRINITY_DN137617_c1_g1	mRNA	2.75
C00735_TRINITY_DN137011_c1_g3	Metabolics	−4.23
C00735_TRINITY_DN137011_c1_g3	mRNA	−10.07
C00735_TRINITY_DN136353_c1_g1	Metabolics	−4.23
C00735_TRINITY_DN136353_c1_g1	mRNA	3.71
C00735_TRINITY_DN139698_c2_g1	Metabolics	−4.23
C00735_TRINITY_DN139698_c2_g1	mRNA	−6.86
C00735_TRINITY_DN124132_c0_g1	Metabolics	−4.23
C00735_TRINITY_DN124132_c0_g1	mRNA	1.46
C00735_TRINITY_DN121491_c0_g1	Metabolics	−4.23
C00735_TRINITY_DN121491_c0_g1	mRNA	−1.17
C00735_TRINITY_DN132364_c1_g1	Metabolics	−4.23
C00735_TRINITY_DN132364_c1_g1	mRNA	−9.68
C00735_TRINITY_DN129390_c5_g2	Metabolics	−4.23
C00735_TRINITY_DN129390_c5_g2	mRNA	−6.67
C00735_TRINITY_DN135404_c0_g1	Metabolics	−4.23
C00735_TRINITY_DN135404_c0_g1	mRNA	6.51
C00881_TRINITY_DN115131_c0_g1	Metabolics	−1.76
C00881_TRINITY_DN115131_c0_g1	mRNA	−1.78
C00881_TRINITY_DN137643_c0_g1	Metabolics	−1.76
C00881_TRINITY_DN137643_c0_g1	mRNA	1.24
C00881_TRINITY_DN135736_c0_g1	Metabolics	−1.76
C00881_TRINITY_DN135736_c0_g1	mRNA	4.02
C01081_TRINITY_DN127457_c1_g1	Metabolics	1.94
C01081_TRINITY_DN127457_c1_g1	mRNA	1.39
C00881_TRINITY_DN126571_c1_g1	Metabolics	−1.76
C00881_TRINITY_DN126571_c1_g1	mRNA	2.59
C01081_TRINITY_DN134733_c0_g2	Metabolics	1.94
C01081_TRINITY_DN134733_c0_g2	mRNA	1.01
C01081_TRINITY_DN127457_c1_g3	Metabolics	1.94
C01081_TRINITY_DN127457_c1_g3	mRNA	1.75
C01081_TRINITY_DN134236_c3_g1	Metabolics	1.94
C01081_TRINITY_DN134236_c3_g1	mRNA	1.13
C01157_TRINITY_DN138437_c1_g1	Metabolics	−1.50
C01157_TRINITY_DN138437_c1_g1	mRNA	5.49
C01157_TRINITY_DN138437_c1_g2	Metabolics	−1.50
C01157_TRINITY_DN138437_c1_g2	mRNA	10.32
C01227_TRINITY_DN133519_c0_g1	Metabolics	−1.11
C01227_TRINITY_DN133519_c0_g1	mRNA	6.67
C01227_TRINITY_DN129783_c0_g3	Metabolics	−1.11
C01227_TRINITY_DN129783_c0_g3	mRNA	2.51
C01227_TRINITY_DN134262_c0_g2	Metabolics	−1.11
C01227_TRINITY_DN134262_c0_g2	mRNA	2.60
C01227_TRINITY_DN134262_c1_g1	Metabolics	−1.11
C01227_TRINITY_DN134262_c1_g1	mRNA	1.93
C01227_TRINITY_DN119253_c3_g1	Metabolics	−1.11
C01227_TRINITY_DN119253_c3_g1	mRNA	5.13
C01227_TRINITY_DN129783_c0_g2	Metabolics	−1.11
C01227_TRINITY_DN129783_c0_g2	mRNA	3.35
C01227_TRINITY_DN134262_c1_g2	Metabolics	−1.11
C01227_TRINITY_DN134262_c1_g2	mRNA	2.24
C01227_TRINITY_DN129528_c2_g1	Metabolics	−1.11
C01227_TRINITY_DN129528_c2_g1	mRNA	−3.03
C01227_TRINITY_DN129783_c0_g1	Metabolics	−1.11
C01227_TRINITY_DN129783_c0_g1	mRNA	2.23
C01227_TRINITY_DN130965_c1_g2	Metabolics	−1.11
C01227_TRINITY_DN130965_c1_g2	mRNA	2.69
C01561_TRINITY_DN122647_c3_g2	Metabolics	1.16
C01561_TRINITY_DN122647_c3_g2	mRNA	10.78
C01561_TRINITY_DN122647_c3_g1	Metabolics	1.16
C01561_TRINITY_DN122647_c3_g1	mRNA	4.93
C01762_TRINITY_DN137643_c0_g1	Metabolics	1.89
C01762_TRINITY_DN137643_c0_g1	mRNA	1.24
C01762_TRINITY_DN135736_c0_g1	Metabolics	1.89
C01762_TRINITY_DN135736_c0_g1	mRNA	4.02
C01762_TRINITY_DN130743_c0_g1	Metabolics	1.89
C01762_TRINITY_DN130743_c0_g1	mRNA	2.43
C01762_TRINITY_DN115131_c0_g1	Metabolics	1.89
C01762_TRINITY_DN115131_c0_g1	mRNA	−1.78
C01879_TRINITY_DN139025_c5_g1	Metabolics	−1.23
C01879_TRINITY_DN139025_c5_g1	mRNA	−2.28
C02029_TRINITY_DN111850_c0_g1	Metabolics	4.18
C02029_TRINITY_DN111850_c0_g1	mRNA	−1.15
C02029_TRINITY_DN128988_c2_g1	Metabolics	4.18
C02029_TRINITY_DN128988_c2_g1	mRNA	1.02
C02137_TRINITY_DN121491_c0_g1	Metabolics	−3.99
C02137_TRINITY_DN121491_c0_g1	mRNA	−1.17
C02137_TRINITY_DN124132_c0_g1	Metabolics	−3.99
C02137_TRINITY_DN124132_c0_g1	mRNA	1.46
C02137_TRINITY_DN135404_c0_g1	Metabolics	−3.99
C02137_TRINITY_DN135404_c0_g1	mRNA	6.51
C05512_TRINITY_DN129874_c1_g1	Metabolics	−2.83
C05512_TRINITY_DN129874_c1_g1	mRNA	5.01
C05512_TRINITY_DN130743_c0_g1	Metabolics	−2.83
C05512_TRINITY_DN130743_c0_g1	mRNA	2.43
C05512_TRINITY_DN125813_c1_g5	Metabolics	−2.83
C05512_TRINITY_DN125813_c1_g5	mRNA	5.81
C05585_TRINITY_DN128233_c1_g1	Metabolics	−5.55
C05585_TRINITY_DN128233_c1_g1	mRNA	3.13
C05585_TRINITY_DN137617_c1_g1	Metabolics	−5.55
C05585_TRINITY_DN137617_c1_g1	mRNA	2.75
C05903_TRINITY_DN111850_c0_g1	Metabolics	1.66
C05903_TRINITY_DN111850_c0_g1	mRNA	−1.15
C05903_TRINITY_DN128988_c2_g1	Metabolics	1.66
C05903_TRINITY_DN128988_c2_g1	mRNA	1.02
C06104_TRINITY_DN139541_c0_g1	Metabolics	−1.12
C06104_TRINITY_DN139541_c0_g1	mRNA	1.53
C06427_TRINITY_DN127483_c1_g1	Metabolics	−2.03
C06427_TRINITY_DN127483_c1_g1	mRNA	2.28
C06427_TRINITY_DN139240_c0_g1	Metabolics	−2.03
C06427_TRINITY_DN139240_c0_g1	mRNA	2.22
C06427_TRINITY_DN119196_c1_g1	Metabolics	−2.03
C06427_TRINITY_DN119196_c1_g1	mRNA	1.04
C06427_TRINITY_DN122469_c1_g1	Metabolics	−2.03
C06427_TRINITY_DN122469_c1_g1	mRNA	1.18
C06427_TRINITY_DN112767_c0_g3	Metabolics	−2.03
C06427_TRINITY_DN112767_c0_g3	mRNA	2.81
C06427_TRINITY_DN137047_c2_g1	Metabolics	−2.03
C06427_TRINITY_DN137047_c2_g1	mRNA	−3.40
C06427_TRINITY_DN110387_c0_g1	Metabolics	−2.03
C06427_TRINITY_DN110387_c0_g1	mRNA	−2.68
C06427_TRINITY_DN119122_c0_g1	Metabolics	−2.03
C06427_TRINITY_DN119122_c0_g1	mRNA	1.97
C15603_TRINITY_DN138437_c1_g1	Metabolics	−7.99
C15603_TRINITY_DN138437_c1_g1	mRNA	5.49
C15603_TRINITY_DN138437_c1_g2	Metabolics	−7.99
C15603_TRINITY_DN138437_c1_g2	mRNA	10.32
C15603_TRINITY_DN123475_c1_g3	Metabolics	−7.99
C15603_TRINITY_DN123475_c1_g3	mRNA	1.15
C15603_TRINITY_DN121542_c0_g4	Metabolics	−7.99
C15603_TRINITY_DN121542_c0_g4	mRNA	3.90
C15658_TRINITY_DN139332_c5_g2	Metabolics	4.40
C15658_TRINITY_DN139332_c5_g2	mRNA	3.29
C17232_TRINITY_DN135404_c0_g1	Metabolics	1.77
C17232_TRINITY_DN135404_c0_g1	mRNA	6.51
C17232_TRINITY_DN121491_c0_g1	Metabolics	1.77
C17232_TRINITY_DN121491_c0_g1	mRNA	−1.17
C17232_TRINITY_DN124132_c0_g1	Metabolics	1.77
C17232_TRINITY_DN124132_c0_g1	mRNA	1.46

**Figure 4:**
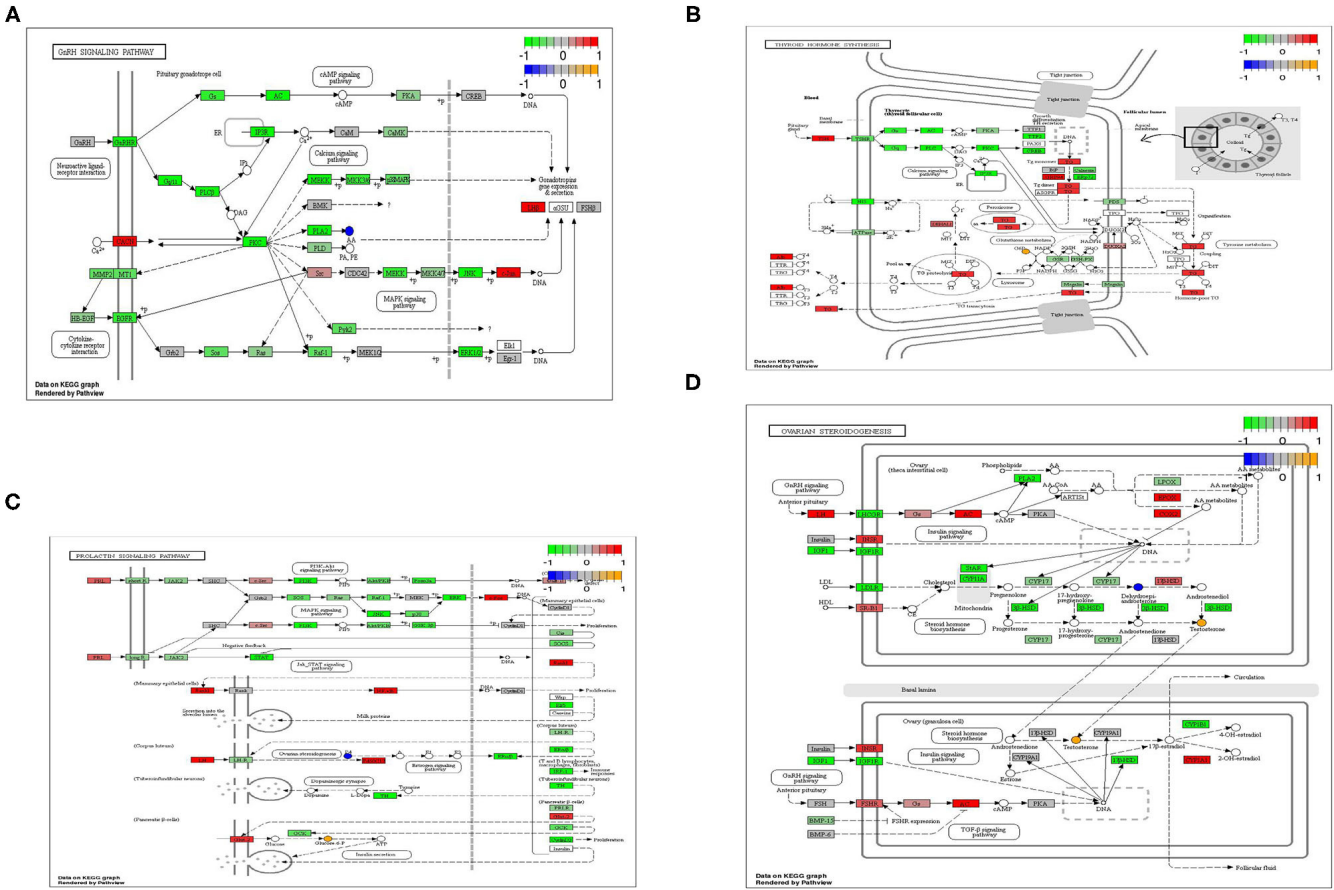
**(A)** GnRH signaling pathway of differential metabolites and differential transcripts. **(B)** PRL signaling pathway of differential metabolites and differential transcripts. **(C)** Thyroid hormone synthesis pathway of differential metabolites and differential transcripts. **(D)** Ovarian steroidogenesis pathway of differential metabolites and differential transcripts.

According to the results of transcripts and metabolites pathway analysis, combined with transcripts and metabolites differential co-expression, there were four metabolic pathways closely relating to light regulation of reproductive performance of the geese has been selected, namely GnRH signaling pathway, prolactin signaling pathway, thyroxine synthesis pathway and ovarian steroid synthesis pathway (Figure 4). In the GnRH signaling pathway, arachidonic acid was a typical differential metabolite, and 36 genes and 112 proteins (transcripts) played corresponding roles in the pathway. The typical differential metabolites involved in the prolactin signaling pathway were glucose-6-phosphate and progesterone, and 15 genes and 67 proteins (transcripts) play corresponding roles in the pathway. In the thyroid hormone synthesis pathway, the typical differential metabolites involved in the hypothalamus were glucose-6-phosphate, while the typical differential metabolites involved in the ovary were glutathione and oxidized glutathione. Meanwhile, 20 genes and 77 proteins (transcripts) played the corresponding roles in the pathway. Arachidonic acid and progesterone were the typical differential metabolites in the hypothalamus, and 24 genes and 77 proteins (transcripts) played corresponding roles in the pathway; The typical differential metabolites involved in the ovary were testosterone and deoxyepiandrosterone, and 24 genes and 69 proteins (transcripts) played corresponding roles in the pathway.

## Discussion

Currently, the widely applied metabonomics research methods include Nuclear Magnetic Resonance (NMR), GC-TOF/MS, and UPLCQ-TOF/MS (14, 15). In comparison, nuclear magnetic resonance (NMR) technology is widely used for its advantages of non-destructive, absolute quantitative, and outstanding repeatability (16). However, meanwhile, its disadvantages are very prominent, such as low detection sensitivity, unable to detect extremely trace and low concentration substances; Although GC-TOF/MS technology can identify more complex metabolites with low cost, the pretreatment is complicated which increases the possibility of errors (17); In recent years, UPLC-Q-TOF/MS technology has been widely used in biomedicine, food science, agricultural science and other research fields by its properties of “separation capability”, “reliability”, and “sensitivity” (18). Through this experiment, UPLC-Q-TOF/MS technology was used to study the metabonomics of the hypothalamus, pituitary, and ovary of Zi-geese under the different light conditions. 18 samples were analyzed, and a quality control sample (QC) was inserted into every 6 samples for quality control to investigate the repeatability of the whole analysis procedure. According to the ion flow diagram of the base peak under the positive and negative ion mode of the quality control sample, the chromatographic peaks were uniform, large in number, good in shape, without oversaturation, tailing, and other adverse phenomena. At the same time, the PCA examination of the test results also showed that all the points of the quality control sample (QC) were closely clustered, which indicated that the whole experimental process has outstanding repeatability and no abnormal data. The results showed that the quality of the test sample was qualified, and the analysis technology was practical and effective. Through the transcriptomic analysis, 1,481 differential metabolites were screened out, among which 583 were screened out from the hypothalamus of Zi-geese and 384 were unique; 196 differential metabolites and 117 unique differential metabolites were screened from the pituitaries of Zi-geese; 692 differential metabolites and 513 unique differential metabolites were screened from the ovary of the geese. The results showed that the changes in light conditions could significantly affect the metabolic processes related to the reproductive performance of Zi-geese.

To further understand the inner mechanism of light regulation on reproductive performance of Zi-geese, the transcriptomics and metabonomics combined analysis technology has been used to analyze the differentially expressed genes and differentially accumulated metabolite information of the test samples for gene-gene, gene-metabolite, metabolite-metabolite “co-expression” analysis, to identify the key metabolic pathway and figure out the main regulatory factors and structural genes. The regulation mechanism was analyzed and verified by physiological, biochemical, genetic, and molecular biology methods. Through transcriptomic and metabonomic analysis, 204 pairs of metabolites were found. The co-expression of transcripts identified four metabolic pathways closely related to the reproductive performance of Zi-geese, which pointed out the direction and goal for understanding the internal mechanism of light regulation of reproductive performance of Zi-geese.

Since the change of light conditions can affect the reproductive performance of poultry and the expression of related genes, the change of light conditions would inevitably lead to the change of metabolites related to reproductive performance. To further reveal the possible mechanism of red light for 12 h improving reproductive performance of Zi-geese, the metabonomics of hypothalamus, pituitary, and ovary samples of Zi-geese were studied. The results showed that 583 differential metabolites were found in the hypothalamus of the geese, and 384 were unique; 196 differential metabolites and 117 unique differential metabolites were screened from the pituitaries of the geese; 692 differential metabolites and 513 unique differential metabolites were screened from the ovaries of Zi Goose. This confirmed that there were significant differences in material metabolism between red light and white light at the peak laying period. Further analysis showed that 433 differentially expressed metabolites were down-regulated and 150 differentially expressed metabolites were u*P-*regulated in the hypothalamus of Zi-geese under red light for 12 h; 125 differential metabolites were down-regulated and 71 differential metabolites were up-regulated; 355 differentially expressed metabolites were down-regulated and 337 differentially expressed metabolites were up-regulated. The amount of these different metabolites might be the main substances that affect the reproductive performance of Zi-geese, and it is also the main object that needs to be focused on in the future. Co-expression of differentially expressed genes and metabolites was confirmed by transcriptome and metabolomic analysis, which provided the guarantee for mining core metabolic pathways and typical metabolites. The results showed that there were 15, 3, and 186 pairs of metabolites in the hypothalamus, pituitaries, and ovaries of Zi-geese. Four metabolic pathways were closely related to the light regulation of reproductive performance in the geese, including the GnRH signaling pathway, prolactin signaling pathway, thyroxine synthesis pathway, and ovarian steroid synthesis pathway. The differential metabolites involved in these metabolic pathways are arachidonic acid, glucose-6-phosphate, progesterone, glutathione, oxidized glutathione, testosterone, and deoxyepiandrosterone, which would play an essential role in the light regulation of the reproductive performance of Zi geese.

## Conclusion

This study was found out the differential metabolites in response to light regulation, and to clarify the regulatory genes of the differential metabolites and their differences in the regulation mode of reproductive performance for the geese. In this study,1,481 differential metabolites were screened, among which 583 differential metabolites were screened from the hypothalamus of seeded goose, and 384 differential metabolites were endemic to the hypothalamus of seeded goose. A total of 196 differential metabolites were screened out from the pituitary gland of the goose, and 117 unique differential metabolites were found. 692 differential metabolites were screened from seed goose egg nests, and 513 differential metabolites were endemic. The accumulation of 433 differential metabolites in the hypothalamus of seed geese decreased under 12 h red light, while the accumulation of 150 differential metabolites increased under 12 h red light. The accumulation of 125 differential metabolites in pituitary gland decreased under 12 h red light condition, while the accumulation of 71 differential metabolites increased under 12 h red light condition. The accumulation of 355 differential metabolites in the nest of seed goose eggs decreased under 12 h red light, while the accumulation of 337 differential metabolites increased under 12 h red light. The results of joint analysis showed that 33 differential metabolites were closely related to the homologous genes of 1,264 transcripts and 400 related enzymes in the hypothalamus of Seed Geese. There were 15 differential metabolites closely related to 163 transcripts and 47 homologous genes of related enzymes in pituitary gland. There were 55 differential metabolites associated with 1,255 transcripts and 360 homologous genes of related enzymes in the nest of seed goose egg. In the hypothalamus, pituitary and ovary of seed geese, 15, 3 and 186 metabolite transcripts were screened out in response to the change of light conditions. Four metabolic pathways were determined to be closely related to light regulation of reproductive performance of seed geese, namely GnRH signaling pathway, prolactin signaling pathway, thyroxine synthesis pathway and ovarian steroid synthesis pathway. Canonical differential metabolites such as arachidonic acid, glucose 6-phosphate, progesterone, glutathione, glutathione oxidase, testosterone and deoxyepandrosterone, as well as their related genes and protein genes, will play an important role in light regulation of reproductive performance of seed geese.

## Data Availability Statement

The original contributions presented in the study are included in the article/supplementary material, further inquiries can be directed to the corresponding author/s.

## Author Contributions

LM: research concept, methodology, data extraction, analysis, and draft writing. ZX: resource searching, verification, formal analysis, supervision, and manuscript reviewing and editing. LG: resources, methodology, project administration, supervision, and manuscript reviewing and editing. ZG: resource searching, manuscript reviewing and editing, and methodology. All authors contributed to the article and approved the submitted version.

## Funding

The research was supported by the research business expenses of the Scientific Research Institutes of Heilongjiang Province (No. CZKYF2021B003) and National Modern Waterfowl Industry Technical System Special Project (No. CARS-42-24).

## Conflict of Interest

The authors declare that the research was conducted in the absence of any commercial or financial relationships that could be construed as a potential conflict of interest.

## Publisher's Note

All claims expressed in this article are solely those of the authors and do not necessarily represent those of their affiliated organizations, or those of the publisher, the editors and the reviewers. Any product that may be evaluated in this article, or claim that may be made by its manufacturer, is not guaranteed or endorsed by the publisher.
